# What are the effects of time‐restricted eating upon metabolic health outcomes in individuals with metabolic syndrome: A scoping review

**DOI:** 10.14814/phy2.70338

**Published:** 2025-05-05

**Authors:** Rory J. Heath, Jessie Welbourne, Daniel Martin

**Affiliations:** ^1^ Peninsula Medical School University of Plymouth Plymouth UK; ^2^ Derriford Hospital University Hospitals Plymouth NHS Trust Plymouth UK

**Keywords:** intermittent fasting, metabolic syndrome, time‐restricted eating, time‐restricted feeding

## Abstract

The primary objective of this scoping review (ScR) was to assess the breadth and type of evidence related to time‐restricted eating (TRE) as an intervention to modify metabolic health outcomes in individuals with diagnosed metabolic syndrome (MetS), a major health challenge due to increasing prevalence and association with other chronic diseases. MetS comprises three or more of hypertension, hypercholesterolaemia, dyslipidaemia, dysregulated glucose homeostasis, and abdominal obesity. TRE, also known as time‐restricted feeding (TRF), restricts food intake to specific time windows within a day, for example, a 10‐h eating period between 10:00 and 20:00. Via multiple mechanisms, TRE interventions may provide an effective tool to prevent and treat metabolic disease such as MetS. While studies have assessed TRE in populations with components of MetS, there is a gap in the knowledge of how effective TRE can be for people with diagnosed MetS. A search of studies published in English in the PubMed (Medline), Embase, Cochrane, and PROSPERO databases was performed in February 2024. Of 3449 articles, 45 underwent full text analysis, and three were accepted into the ScR. These studies, comprising 10 and 8 h TRE interventions for 12 weeks, showed mixed benefits to body composition markers such as body weight, fat mass, and abdominal fat, blood pressure, and blood markers of lipid and glucose homeostasis. Future research into TRE and MetS will aim to more closely define optimal formulations of TRE interventions to improve MetS and its components.

## 1 INTRODUCTION

### 1.1 Metabolic syndrome

First defined in 1988 as “Syndrome X” (Reaven, 1988), several definitions of metabolic syndrome (MetS) now exist, each reflecting a constellation of hypertension, abdominal obesity, and abnormal glucose, lipid, and cholesterol metabolism (Alberti et al., 2009; Grundy et al., 2004; Kassi et al., 2011). The presence of MetS confers an increased risk of atherosclerotic cardiovascular disease and type 2 diabetes mellitus (Grundy et al., 2005), and other diseases including neurodegenerative diseases (Fakih et al., 2022; Luque‐Contreras et al., 2014) and cancers (Cao et al., 2020). NHANES data demonstrate increasing prevalence of MetS in the United States, affecting 37.6% of the population in 2011–2012 to 41.8% in 2017–2018 (Liang et al., 2023).

### 1.2 Intermittent fasting, time‐restricted eating, and time‐restricted feeding

Intermittent fasting (IF) is a popularly used term to describe intermittent caloric restriction but encompasses many different arrangements of feeding and fasting windows. The term IF commonly describes longer periods of fasting achieved by alternating days of feeding and fasting (alternate day fasting (ADF)), by fasting for 2 days per week (e.g., the 5:2 diet), or 1 week per month, or prolonged fasting for religious reasons.

In contrast, “time restricted eating” (TRE) and “time restricted feeding” (TRF) describe eating and fasting during defined times on a daily basis, with the feeding period often being shorter than the fasting period (Chaix et al., 2019; Fanti et al., 2021). The distinction between TRE and TRF has been made whereby TRE is applied to humans and TRF is applied to animals in a laboratory setting (Chaix et al., 2019); in this review, the term TRE will be used. A popular example of TRE is of fasting within a 16 h window and eating during the remaining 8 h, a pattern often termed 16:8, while other arrangements (e.g., 12:12, 10:14, etc.) can exist. TRE emphasizes modification of the timing of food intake which may confer independent health and metabolic benefits, while food quality and caloric intake are not necessarily changed (Regmi & Heilbronn, 2020). TRE presents an attractive intervention due to its simplicity, in contrast to pharmacological or exercise interventions that are reliant on factors such as medications or physical ability with their associated financial costs. Furthermore, TRE may provide synergistic effects with pharmacological, dietary, and exercise interventions. As such, TRE may provide an affordable, accessible, and impactful tool for the increasing population of people with MetS, thus its effectiveness should be investigated.

### 1.3 Potential mechanisms of benefit of TRE

#### 1.3.1 Circadian rhythms, health, and disease

The endogenous circadian rhythm spans approximately 24 h (circa‐dian, approximately 1 day) (Panda, 2016). The “central” or “master” clock in the suprachiasmatic nucleus (SCN) of the brain is accompanied by peripheral clocks in virtually every organ, tissue and cell. Light and dark cycles, thus day and night cycles, are detected by intrinsically photosensitive retinal ganglion cells and relayed via the retinohypothalamic tract to the SCN (Hannibal et al., 2004). The SCN coordinates the peripheral clocks via neuroendocrine signaling, such as the diurnal variation in secretion of hormonal mediators such as melatonin, glucocorticoids, and growth hormone. In humans, activity and eating occurs during the daytime or light‐cycle, in contrast to nocturnal species such as rodents.

There is growing understanding that to maintain health the circadian clocks in all tissues must be aligned; as they have been throughout evolution of humans and animals alike with feed‐fast, light–dark, and wake–sleep cycles (Brainard et al., 2015). Without coordination by the SCN, clocks in peripheral tissues function independently and can be influenced by external factors, such as physical activity, food and temperature, that act as circadian cues or “zeitgebers” (time‐givers). Zeitgebers may encourage alignment or misalignment of central and peripheral clocks (Gamble et al., 2014; Mohawk et al., 2012). Disruption of feeding times and light–dark cycles can uncouple the peripheral clocks from the SCN (Damiola et al., 2000), and is associated with metabolic dysfunction and diseases such as obesity and type 2 diabetes (Mason et al., 2020; Saran et al., 2020).

#### 1.3.2 TRE synchronizes energy intake with circadian biology

TRE protocols may restore synchrony between peripheral and central clocks to improve metabolic health via prevention of caloric intake at circadian‐inappropriate times. For example, mice fed a high fat diet ad‐libitum gain weight, while mice provided food overnight (i.e., circadian‐appropriate feeding) within a 9‐h TRE window gain less weight, despite ingesting a similar number of calories (Olsen et al., 2017). In humans, TRE protocols of short duration (most <8 weeks) are associated with reduced triglycerides, lipids, inflammatory markers, blood pressure, and glucose control (Regmi & Heilbronn, 2020). Similarly, in men at risk of type 2 diabetes, 9‐h TRE improved glucose tolerance and fasting triglycerides (Hutchison et al., 2019).

#### 1.3.3 Fasting physiology

Fasting promotes a metabolic switch from dietary‐derived glucose to adipose tissue‐derived free fatty acids and ketone production, which can occur 8–16 h after fasting; thus, it is possible with TRE and IF (Anton et al., 2018). Fasting promotes organ‐specific changes to improve flexibility of fuel utilization, fatty acid and ketone oxidation, insulin sensitivity, and mitochondrial biogenesis, in contrast to obesity‐induced mitochondrial dysfunction (Anton & Leeuwenburgh, 2013; Mendrick et al., 2018). Ketones are potent signaling molecules acting upon myriad pathways, including those relevant to health and aging (Newman & Verdin, 2017).

#### 1.3.4 Aims

This scoping review aims to:

1. determine the effects of time‐restricted eating on metabolic health outcomes in individuals with metabolic syndrome
2. generate questions that will guide future research.

## 2 METHODS

The scoping review of the literature was performed using an interpretive scoping literature review methodology, informed by the Joanna Briggs Institute's methodology for scoping reviews (Peters et al., 2020). The Preferred Reporting Items for Systematic reviews and Meta‐Analyses (PRISMA) extension for Scoping Reviews checklist was utilized (Tricco et al., 2020).

The first stage was to assess novelty, to define a search string of keywords, and to describe inclusion and exclusion criteria. The second stage of review comprised data processing and extraction. The third stage was the reporting of results and synthesis of discussion for future work.

Prospective searches of the OSF, Joanna Briggs Institute (JBI), and PROSPERO databases were performed to ensure novelty and to prevent duplication of research efforts. No ongoing review protocols or systematic reviews with meta‐analysis were found. In this way, to justify the conduct of this research and with a view to clarify the transparency of the construction process and increasing the methodological rigor, as well as the quality of the combined results, it was decided to publish this systematic review protocol.

A search string including MeSH terms was predefined with the guidance of a university‐based medical information specialist. An initial limited search of PubMed (MEDLINE) was performed to guide the construction of the search string using terms within titles and/or abstracts and indexing terms including MeSH terms. A systematic search of PubMed (MEDLINE), Embase, Cochrane, and PROSPERO databases was performed, including all papers published until the 15th of February 2024. Reference lists of included articles were hand‐searched for further relevant articles. Key search terms were metabolic syndrome, intermittent fasting, time‐restricted eating, time‐restricted feeding, and fasting. Full search strategies used for each database are included in the Appendix.

### 2.1 Study eligibility

Inclusion criteria:

- Human studies.
- English language.
- Publication date until February 2024.
- Interventional trials of TRE‐interventions occurring on a daily basis.
- Comparison to normal diet or another dietary pattern.
- Adults with diagnosed MetS.
- Outcomes relevant to metabolic health.

Exclusion criteria:

- Animal studies.
- Abstracts, posters, and protocols.
- Studies of fasting periods >24 h.
- Studies where the feeding window occurs during the astrological night time or inactive period, including those studies of religious fasts.

### 2.2 Data processing and extraction

Search results from each database were downloaded onto a computer and uploaded onto the Rayyan online screening application (Ouzzani et al., 2016). Article inclusion and exclusion were determined by two reviewers (RH and JW) working independently. Conflicts were resolved by consensus. A Microsoft Excel spreadsheet utilizing fields described within the previously published protocol was used as a data collection tool (Microsoft Corporation, 2018).

## 3 RESULTS

**FIGURE 1:**
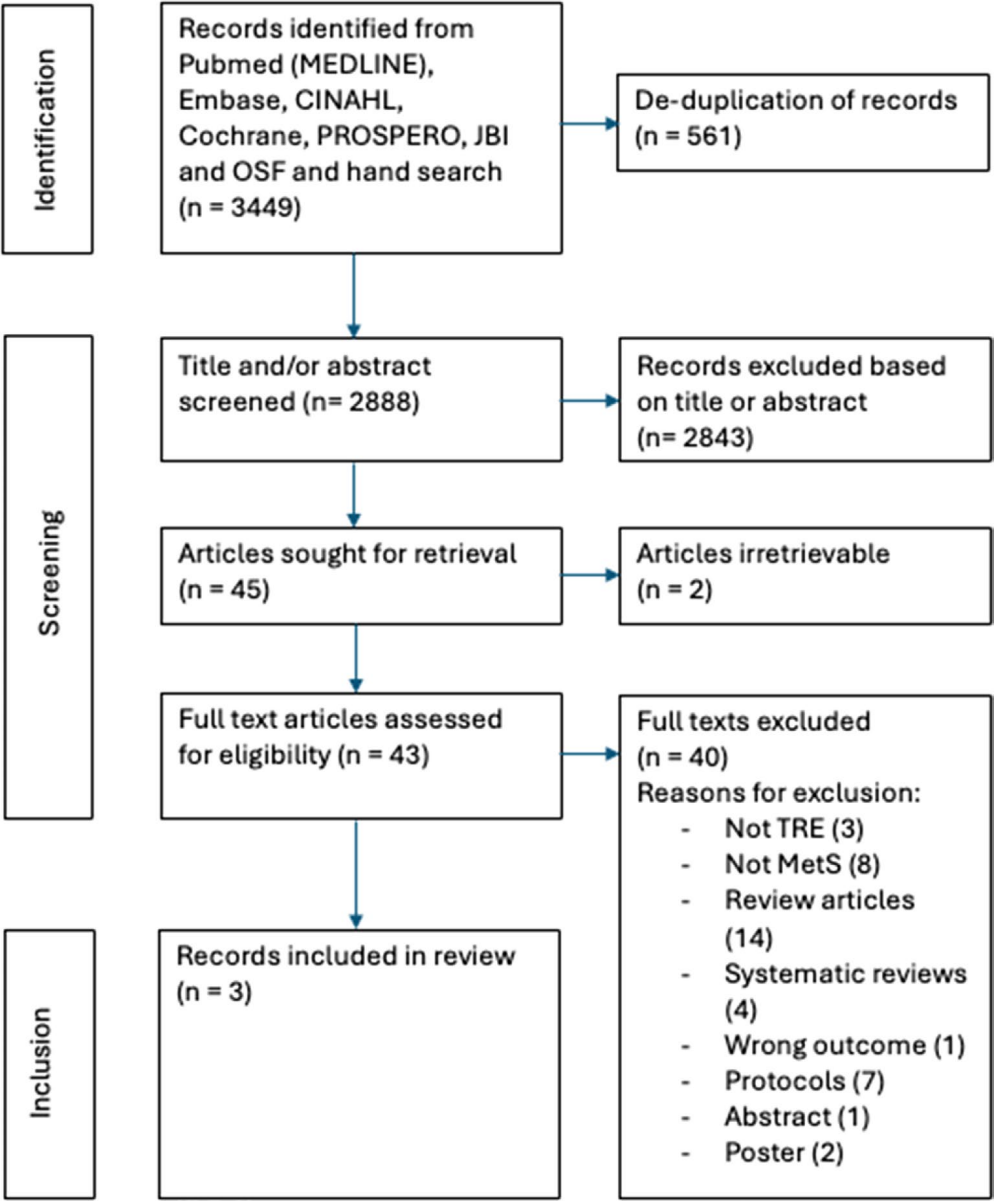
Inclusion decision flowchart.

The search identified a total of 3449 articles, with 2888 remaining after 561 duplicates were removed. Two researchers (RH and JW) independently analyzed and agreed to exclude 2843 articles by comparing titles and abstracts against inclusion criteria. Forty‐five articles underwent full text analysis. Of those, 40 articles were subsequently rejected, and two could not be retrieved. No new articles were identified within the references of these articles. The inclusion decision process is demonstrated in Figure 1. Key findings are summarized in Table 1.

**TABLE 1:** Summary table of included articles.

Reference	Study type	Participants	Intervention	Duration of intervention	Comparator	Metabolic outcomes	Statistically significant results
Wilkinson et al., (2020)	Single arm paired‐sample	Adults with MetS diagnosed using the AHA/NHLBI criteria *N* = 25 Mean age 59 years	10‐h TRE Self‐selected eating window, unrecorded	2 weeks baseline period 12 weeks intervention	Pre‐ and post‐ intervention change measured for each individual (paired sample) and described as mean values for the group.	Anthropometry: BW, WC, WHR, BF%, BMI, VFR, SBP, DBP. Glycaemic control: Mean blood glucose (CGM), FBG, HbA1c, fasting insulin, HOMA‐IR. Lipids and inflammation: HDL‐C, LDL‐C, LDL‐p, non‐HDL‐C, TG, TC, hs‐CRP, ALT, AST.	Cardiometabolic factors: BW (−3%, *p* < 0.001) BMI (−3%, *p* < 0.001) BF% (−3%, *p* < 0.001) WC (−4%, *p* = 0.009) VFR (−3%, *p* = 0.004) SBP (−4%, *p* = 0.041) DBP (−8%, *p* = 0.004) Lipids: TC (−7%, *p* = 0.03) LDL‐C (−11%, *p* = 0.016) Non‐HDL‐C (−9%, *p* = 0.04)
He et al. (2022)	RCT	Adults with MetS. Diagnostic criteria available in original study Participants (*n*) LCD = 55 TRE = 55 LCD + TRE = 52 Of which (*n*): eTRE = 70 lTRE = 37 Mean age = 41	8‐h TRE or 8‐h TRE + LCD Self‐selected eating window, recorded	2 weeks baseline period 12 weeks intervention	LCD ad‐libitum. Post‐hoc exploratory analysis of individuals following patterns of early TRE (eTRE) between 07:00–15:00 or late TRE (lTRE) between 12:00 and 20:00	Anthropometry: BW, BMI, SFA, VFA, SBP, and DBP. Glycaemic control: FBG, HbA1c, HOMA‐IR, HOMA‐IS, fasting insulin, c‐peptide. Plasma lipids: TC, TG, LDL‐C, HDL‐C, TG/HDL‐C ratio. Other: UA	TRE pre‐post comparison: BW (−3.4 kg, *p* < 0.001) VFA (−13 cm^2^, *p* = 0.008) BMI (−1.4 kg/m^2^, *p* < 0.001) TRE + LCD pre‐post comparison: BW (−5 kg, *p* < 0.001) VFA (−10 cm^2^, *p* = 0.006) BMI (−1.8 kg/m^2^, *p* < 0.001) TRE vs LCD, at 3 months: Reduced BW (TRE −3.4, LCD −2.2, *p* −0.013). Reduced WHR (TRE −0.04, LCD –0.01, *p* = 0.023). Greater loss of muscle mass (TRE −0.5 kg, LCD 0.1, *p* < 0.048) VFA (TRE −13 cm^3^, TRE 6 cm^3^, *p* = 0.009)
Kunduraci & Ozbek, (2020)	RCT	Adults with MetS diagnosed by IDF 2005 or NCEP‐ATP III criteria *N* = 70 (M 31/F 34). Mean age 48	8‐h TRE with 25% CR Self‐selected eating window, unrecorded.	12 weeks intervention	Pre‐ and post‐intervention comparison and comparison against 25% CR	Anthropometry BMI, WHR, BP, BF%, FM, FFM, TBW, SBP, DBP, TWL. Glycaemic control: FBG, HbA1c, fasting insulin, and insulin resistance, HOMA‐IR Plasma lipids: TC, LDL‐C, HDL‐C, TG Other: ALT, AST	TRE pre‐post comparison: BW (−8.27, *p* < 0.001) FM (5.52 kg, *p* < 0.001) BMI (−3.06 kg/m^2^, *p* < 0.001) FFM (−2.75 kg, *p* < 0.001) WHR (−0.04, *p* < 0.01) SBP (−7.35 mmHg, *p* < 0.001) DBP (−4.75 mmHg, *p* < 0.001) LDL (−17 mg/dL, *p* < 0.001) TC (−29.32, *p* < 0.001) TG (−41.84, *p* < 0.001) FBG (−15.47 mg/dL, *p* < 0,001) HOMA‐IR (−1.29, *p* < 0.001) HbA1c (−0.32%, *p* < 0.001) Intergroup (TRE vs. CR) comparison: Statistically significant reductions in BMI (TRE −3.06 kg/m^2^, CR −2.13 kg/m^2^, *p* = 0.001) BW (TRE −8.27 kg, CR −5.81 kg, *p* < 0.001) TWL (TRE −8.27 kg, CR −5.8 kg, *p* = 0.020) WC% (TRE −8%, CR −6%, *p* = 0.04) WC (cm) (TRE −6.84 cm, CR −5 cm, *p* = 0.04)

Three articles were included (He et al., 2022; Kunduraci & Ozbek, 2020; Wilkinson et al., 2020), with a total of 246 individuals, of which 100 were female (40%). The mean age of participants across all three interventional studies was 49 years. Studies took place in the USA, China, and Turkey. Studies were designed as RCTs or as a single‐arm paired sample design (pre‐ post‐ intervention comparison).

### 3.1 Participants

All studies included patients with a diagnosis of MetS. The AHA/NHLBI, IDF 2005, NCEP‐ATP definitions of MetS were used. He et al. (2022) used diagnostic criteria that resembled no single consensus definition but that shared similarities with all definitions. Kunduraci et al. specified that participants must have a body mass index (BMI) >27 kg/m^2^.

Wilkinson et al. (2020) specified participants with MetS with a pre‐intervention eating window >14 h. Patients were recruited from hospital‐based clinics (Wilkinson et al., 2020) such as after a clinician referral to a diet clinic (Kunduraci & Ozbek, 2020), while no information regarding the recruitment origin was described by He et al. (2022).

### 3.2 Intervention and comparison

Eating windows were defined in study protocols as either 8 or 10 h. Wilkinson et al. (2020) reduced eating windows from >15 h to 10 h. He et al. restricted the eating window from a baseline duration of 10 h to 8 h, but the average eating window in the TRE groups was 6.5 h. Kunduraci & Ozbek, (2020) defined an 8‐h TRE window but did not record the actual eating window that participants adhered to.

All studies allowed participants to choose the time of onset of their eating window. Kunduraci & Ozbek, (2020) and Wilkinson et al. (2020) did not present these data, while He et al. (2022) separated and analyzed participants as “early” or “late” TRE (eTRE, lTRE) defined as being between 0700 and 1500 or 1200 and 2000, respectively.

Intervention periods in all studies were 12 weeks. Wilkinson et al. (2020) and He et al. (2022) utilized baseline periods of 2 weeks to collect pre‐intervention data totaling a 14‐week study period.

TRE was the sole intervention in one study (Wilkinson et al., 2020) and compared against baseline habits including a normal diet. In other studies, TRE with 25% CR was compared against 25% CR alone (Kunduraci & Ozbek, 2020). Finally, TRE was compared against an ad libitum low‐carb diet (LCD) group and a TRE with LCD group as pairwise comparisons, and all interventions were compared against baseline (He et al., 2022).

### 3.3 Outcomes measures

Measurements were taken at baseline, with studies taking interval measurements at 1, 2, 4, 8, 12, 13, and 14 weeks. Primary outcomes included body weight, abdominal fat area, changes to MetS components, or were undefined.

#### 3.3.1 Anthropometric data

All studies measured total body weight (TBW), BMI and body fat, systolic blood pressure (SBP), and diastolic blood pressure (DBP). Other measures included waist and hip circumference (WC, HC), waist: hip ratio (WHR), body fat mass, body muscle mass, fat‐free mass (FFM), total body weight (TBW), abdominal fat area (AFA), subcutaneous fat area (SFA), and visceral fat area (VFA) or visceral fat rating (VFR). Body composition data was universally assessed using bioelectrical impedance analysis.

#### 3.3.2 Metabolic health

All studies included measures of total cholesterol (TC), high‐ and low‐density lipoprotein cholesterol (HDL‐C, LDL‐C), and triglycerides (TG). He et al. (2022) calculated the TG:HDL‐C ratio, while Wilkinson et al. (2020) included low‐density lipoprotein particle number (LDL‐P), non‐HDL‐C, and high‐sensitivity C‐reactive protein (hs‐CRP). Wilkinson et al. (2020) and Kunduraci & Ozbek, 2020 measured alanine transferase (ALT) and aspartate transferase (AST).

All studies measured fasting glucose, fasting insulin, homeostatic model of assessment of insulin resistance (HOMA‐IR), and glycosylated hemoglobin A1c (HbA1c). He et al. (2022) included measures of C‐peptide, homeostatic model of assessment of insulin sensitivity (HOMA‐IS), quantitative insulin sensitivity check index (QUICKI), and uric acid. Wilkinson et al. (2020) included continuous glucose monitor (CGM) derived average glucose.

#### 3.3.3 Dietary variables

Wilkinson et al. (2020) and He et al. (2022) measured the daily eating interval, using a smartphone app or a food diary, respectively. Kunduraci & Ozbek, 2020 used food diaries and fasting logbooks. Wilkinson et al. (2020) and Kunduraci & Ozbek, 2020 measured caloric intake, while He et al. (2022) measured only carbohydrate intake. Kunduraci & Ozbek, 2020 measured all dietary macronutrients.

#### 3.3.4 Other measures

Wilkinson et al. (2020) measured daily activity using actigraphy, as well as measures of sleep duration and sleep quality (Pittsburgh Sleep Quality Index, PSQI), in addition to thyroid stimulating hormone (TSH), white blood cell, hemoglobin concentration, and platelet count. Kunduraci & Ozbek, 2020 utilized the Physical Activity Questionnaire Short Form.

### 3.4 Feasibility

Wilkinson et al. demonstrated that a TRE intervention is feasible, resulting in a reduction in the eating window from a baseline of 15 h to 10 h, associated with a spontaneous caloric reduction of 8.62% ± 14.47%. The TRE and TRE + LCD groups in He et al. (2022) reduced the eating window from a baseline of 10 h to a post‐intervention duration of 6.5 and 6.8 h, respectively. The TRE group had 98% willingness to continue the dietary intervention.

Attrition of participants during the intervention period was 7%, 16%, and 24%. Reasons included low adherence to diet, poor data logging by individuals, loss to follow up, scheduling conflicts, need for surgery, or pregnancy. No study reported any serious adverse events. Only He et al. reported negative patient experiences (constipation, dizziness, insomnia, dry mouth, and alopecia).

Two studies reported adherence to their respective interventions. Wilkinson et al. (2020) reported adherence to data logging of 85% and adherence to meal windows on 93% of days. He et al. (2022) reported adherence to meal timing interventions on 64%–73% of days during the intervention period. Adherence for individuals in the eTRE group was 68% and lTRE 83%. Kunduraci & Ozbek, 2020 described a loss of 7% of participants; however, those entering analysis in either the TRE + CR or CR group reduced their caloric intake by 25%.

### 3.5 Efficacy

All articles demonstrated the benefit of TRE in comparison to baseline with regards to anthropometric measures. Wilkinson et al. (2020) found that TRE reduced body weight, BMI, body fat percentage, WC, VFR, SBP, and DBP. Kunduraci & Ozbek, 2020 demonstrated that TRE + CR reduced weight, fat mass, FFM, TBW, BMI, WHR, SBP, and DBP. He et al. (2022) found that TRE reduced TBW, BMI, WC, HC, WHR, fat mass, muscle mass, SFA, and VFA, but did not find effects on SBP or DBP.

All groups found beneficial changes to blood markers of metabolic health in comparison to baseline. With regard to lipids, Wilkinson et al. (2020) found that TRE reduced TC, LDL‐c, and non‐HDL‐c. No significant changes were seen to LDL‐p, HDL‐c, or TGs. Kunduraci et al. found significant beneficial effects of TRE + CR to LDL‐c, TC, TG, but no effect was seen regarding HDL‐c. He et al. (2022) found no significant change to total cholesterol, LDL‐c, HDL‐c, or HbA1c. With regard to glucose homeostasis, Wilkinson et al. (2020) did not find significant changes to fasting blood glucose or CGM‐derived average glucose. Kunduraci & Ozbek, 2020 found significant benefits to HOMA‐IR, fasting glucose, and HbA1c, but no benefit to fasting insulin. He et al. (2022) showed that the TRE and TRE + LCD groups improved fasting blood glucose, fasting insulin, c‐peptide, HOMA‐IR, HOMA‐IS, and QUICKI. Regarding other metabolic health markers, Wilkinson et al. (2020) found significant reductions in uric acid, but no significant changes in hs‐CRP, ALT, or AST. He et al. (2022) found significant changes to uric acid.

## 4 DISCUSSION

Of 3449 retrieved articles, only three met the inclusion criteria. These articles comprised two RCTs and one pre–post comparison study, included few patients from different populations and countries, used differing diagnostic criteria, and shared no consistent comparator. These findings clearly indicate the sparsity of available evidence assessing the effects of TRE on metabolic outcomes in individuals with MetS. This article sought to identify the effects of TRE on metabolic health outcomes, finding that the outcomes studied were of the many parameters used to diagnose MetS and characterize its components. Of wider importance, no outcomes measured clinically meaningful and patient‐centric endpoints such as reduced insulin requirements, fewer hospital admissions, weight loss, or measures of longevity. We may not yet know which outcome is best to measure the global effects of TRE upon metabolic health.

We may consider assessing TRE protocols by modifying a tool used to define physical exercise protocols, the “FITT” tool (ACSM, 2010). The "FITT" tool defines interventions by frequency, intensity, time and type, where “frequency” can represent how often the TRE intervention is required, “intensity” may represent the degree of caloric restriction, and “timing” may represent the duration of the fast. “Type” may represent additional dietary changes, for example, a low‐carbohydrate diet, or positioning of the fasting window within the day, for example, early or late TRE.

### 4.1 Frequency

Within these studies, the optimal frequency of TRE interventions is not explored. Future work may aim to understand whether TRE is required daily or whether fewer TRE days can provide metabolic benefits.

### 4.2 Intensity

It is yet unknown whether the effects of TRE are separate from the effects of CR. Kunduraci & Ozbek, 2020 defined a large caloric deficit (25%) while He et al. did not define or report any deficit, yet both groups demonstrated changes to different measured parameters of MetS. It is unknown whether these effects of TRE and CR are synergistic. Kunduraci & Ozbek, 2020 demonstrated that body weight and waist circumference were more greatly reduced in the TRE + CR group than in CR alone despite equal caloric deficits.

### 4.3 Timing

Within these studies, Wilkinson et al. (2020) reduced the eating window from >15 h to 10 h and showed no changes to fasting glucose or HOMA‐IR, while participants of He et al. (2022) showed benefit with a reduction from baseline 10 h to 6 h. On the other hand, 6 h may be unnecessary; baseline measurements of blood pressure and total cholesterol in participants of He et al. (2022) were within the normal range.

### 4.4 Type

TRE may make circadian rhythms more robust via eating at circadian‐appropriate times (Tippairote et al., 2021), there may be benefit to positioning the eating window at different times. For example, He et al. (2022) reported greater reductions in VFA in the eTRE versus lTRE group. These effects may be uncovered by the registered clinical trial (NCT06018415), comparing the effects of early and late time‐restricted feeding on overweight adults with metabolic syndrome. In addition, other factors may alter the efficacy of TRE to improve metabolic outcomes, for example, concurrent dietary changes such as the low‐carbohydrate diet.

### 4.5 Methodological limitations of this ScR

Despite a broad literature search encompassing both keywords and MeSH terms, designed in conjunction with a university‐based medical information specialist, it is possible that the search missed studies that did not use these terms. Indeed, the MeSH term “Intermittent Fasting” was introduced in 2023 and there is not yet a term for TRE; it is possible that studies published before this time used other terminology. Furthermore, this ScR excluded studies published in non‐English languages, and those presented as abstracts, posters, or in published in the gray literature.

### 4.6 Future research

Our knowledge regarding the optimal formulation of TRE interventions to improve metabolic syndrome is limited. Future work should aim to better understand how manipulating TRE variables in terms of frequency, intensity, time, and type may alter effects, to better understand the dose–response effect of TRE, to elicit differences in TRE window timing, and to separate the roles of TRE and CR. We should seek to define and measure outcomes considered meaningful to patients and clinicians.

## 5 CONCLUSION

The literature relevant to TRE interventions in patients with MetS is sparse but describes a consistent benefit to clinically important anthropometric measures and blood biomarkers related to MetS.

## AUTHOR CONTRIBUTIONS

RH and DM conceived the article; RH and DM designed the study protocol; RH and JW undertook the study; RH drafted the initial manuscript; RH and DM critically revised the manuscript. All authors read and approved the final manuscript.

## FUNDING INFORMATION

There are no sources of funding for this work. R.H. holds an NIHR‐funded Academic Clinical Fellow post in Anaesthesia.

## CONFLICT OF INTEREST STATEMENT

No conflicts of interest are declared.

## Data Availability

The data supporting the findings of this article are available within the article and its Appendix.

## EMBASE: EXCERPTA MEDICA (OVID)

((“intermittent fasting” or “Time restricted eating” or “Time restricted feeding”).af OR (exp intermittent fasting/ or fasting/)) AND ((“metabolic syndrome”.af.) OR (metabolic syndrome X/))

## COCHRANE LIBRARY

((MeSH descriptor: [Fasting] explode all trees) OR (“Intermittent fasting” OR “Time restricted eating” OR “time restricted feeding”)) AND ((MeSH descriptor: [Metabolic Syndrome] explode all trees) OR “metabolic syndrome”)

## PUBMED (MEDLINE)

(“metabolic syndrome”[All Fields] OR “metabolic syndrome”[MeSH Terms]) AND (“intermittent fasting”[All Fields] OR “Time restricted eating”[All Fields] OR “time restricted feeding”[All Fields] OR “Fasting”[MeSH Terms])

## PROSPERO

(MeSH DESCRIPTOR Fasting EXPLODE ALL TREES OR “intermittent fasting” OR “time restricted eating” OR “time restricted feeding” OR “fasting”) AND (MeSH DESCRIPTOR Metabolic Syndrome EXPLODE ALL TREES OR “metabolic syndrome”)

## OSF

(“intermittent fasting” OR “fasting” OR “Time restricted eating”) AND (“metabolic syndrome”)
